# One-year prospective clinical study comparing patient satisfaction and masticatory performance of mandibular overdentures supported by one versus two implants

**DOI:** 10.1590/1678-7757-2016-0628

**Published:** 2018-10-02

**Authors:** André Gustavo Paleari, Norberto Martins de Oliveira, Danny Omar Mendoza Marin, Larissa Santana Rodriguez, João Neudenir Arioli, Ana Carolina Pero, Marco Antonio Compagnoni

**Affiliations:** 1 Universidade Federal de Alfenas, Faculdade de Odontologia, Departamento de Odontologia Restauradora, Alfenas, Minas Gerais, Brasil.; 2 Univ. Estadual Paulista, Faculdade de Odontologia de Araraquara, Departamento de Materiais Odontológicos e Prótese, Araraquara, São Paulo, Brasil.

**Keywords:** Dental implants, Dentures, Patient satisfaction

## Abstract

There is a lack of clinical evidence on mandibular overdentures (MOD) supported by a single implant.

**Objective:**

To compare patient satisfaction and masticatory performance in MOD supported by one versus two implants in a two-group parallel randomized clinical trial.

**Material and Methods:**

Twenty-one patients wearing new maxillary and mandibular complete dentures (CDs) were randomly divided to receive one (GI, n = 11) or two (GII, n = 10) implants in the mandibular arch. Four months after implant placement, o-ring abutments were installed in the implants, and matrix attachments were placed in the lower complete dentures. Patient satisfaction with their dentures and masticatory performance were compared at baseline, 3, 6, and 12 months after the procedure. Data on patient satisfaction were analyzed using the Friedman test and the Mann-Whitney U test. Data on masticatory performance were analyzed using one-way repeated measures analysis of variance (ANOVA) and Student's t test (α=0.05).

**Results:**

Both groups exhibited a significant increase in overall patient satisfaction in all periods evaluated (p<0.05), except for GI after 12 months, which had values similar to baseline (p=0.74). Satisfaction levels of GI and GII were similar at baseline, 3 and 6 months, but GII showed higher satisfaction levels (p=0.01) than GI at 12 months. GI and GII exhibited a significant increase (p<0.05) in masticatory performance for all periods relative to baseline. However, GII had higher masticatory performance with dentures than GI, regardless of the period (p<0.05).

**Conclusion:**

MOD supported by two implants demonstrated better patient satisfaction in the follow-up at 12 months and better masticatory performance than MOD supported by one implant.

## Introduction

The lack of retention and stability of mandibular complete dentures (CDs) often result in several problems for edentulous patients, as mastication issues, decreased quality of life and self-confidence, reduced satisfaction, and more limited social contact ^1^ . According to MacEntee ^2^ (2003), complete dentures supported by implants can offer comfort, optimism, and relief for these patients.

It is known that mandibular overdentures (MOD) supported by two implants exhibit good long-term results and are commonly proposed for treatment of edentulous mandibles ^3^ . However, no robust evidence has been found to support this treatment as a single standard of care for patients with an edentulous mandible ^4^ , since the costs are prohibitive for most edentulous patients ^5^ ^,^ ^6^ and there is a difficulty in placing the implants in reduced buccolingual dimensions of the bone without using bone graft procedures ^7^ .

Recent prospective studies have suggested the use of a single implant to support a MOD ^5^ ^,^ ^6^ ^,^ ^8^ ^-^ ^14^ . This modality is originally recommended for older edentulous patients whom experience discomfort and functional difficulties with their conventional mandibular dentures ^10^ . One implant is inserted in the midline symphysis region of the mandible after adequate presurgical diagnoses and is used for anchorage of the mandibular overdenture ^12^ . This less invasive and less costly intervention could allow more people to benefit from this treatment, even with general health concerns ^13^ .

In a recent systematic review and meta-analysis, Srinivasan, et al. ^6^ (2016) affirmed that the existing scientific evidence on MOD supported by a single implant is still scarce. Consequently, before recommending this treatment for edentulous mandibles, long-term observations are needed, and larger functional and patient-centered outcome studies are suggested ^6^ .

Patient satisfaction is one factor that influences the success of overdenture treatment ^7^ . Previous studies have demonstrated that MOD supported by a single implant significantly improves patient satisfaction in comparison to a conventional mandibular CD ^5^ ^,^ ^15^ , but scientific evidence regarding satisfaction of the MOD supported by one versus two implants is scarce in the literature ^5^ ^,^ ^9^ ^,^ ^14^ ^,^ ^16^ .

Masticatory performance is an important index for measuring patients’ oral function, as well as the effectiveness of their prosthesis ^15^ . According to Boven, et al. ^17^ (2015), the treatment of patients with CD implants to support their denture improves their chewing efficiency and increases the maximum bite force. However, there seem to be no articles in the literature to date that compare masticatory performance between MOD supported by one versus two implants.

The objective of this study was to compare patient satisfaction and masticatory performance in MOD supported by one versus two implants after one year in a prospective clinical study. The null hypothesis was that patient satisfaction and masticatory performance would not differ among groups in the follow-up at 12 months, regardless of the number of implants.

## Material and methods

### Study design

This two-group parallel randomized clinical trial was conducted at the graduate clinic of the Department of Prosthodontics, School of Dentistry, Univ. Estadual Paulista (UNESP), Araraquara-SP, Brazil. The study was approved by the Institutional Ethics Committee (CAAE No. 02818012800005416) and registered in the Brazilian Clinical Trials Registry (ReBEC - trial number: RBR-5frcz9).

### Study population

Individuals who had been referred to the graduate clinic were assessed for eligibility according to the following inclusion criteria: complete edentulism in maxillary and mandibular arches for at least one year; desire to receive new CDs and to replace mandibular CDs with an overdenture; minimum bone height of 15 mm in the mandibular edentulous ridge examined on panoramic radiographs; normal resilience of residual mucosa (displacement of approximately 2 millimeters), assessed by a clinician; and good overall health. The exclusion criteria were: parafunctional habits or any movement limitations that may interfere with the chewing test; previous treatment with implants; uncontrolled diabetes mellitus; alcoholism or smoking habit; poor systemic health; impossibility to return to recall appointments or follow-up visits.

**Figure 1:**
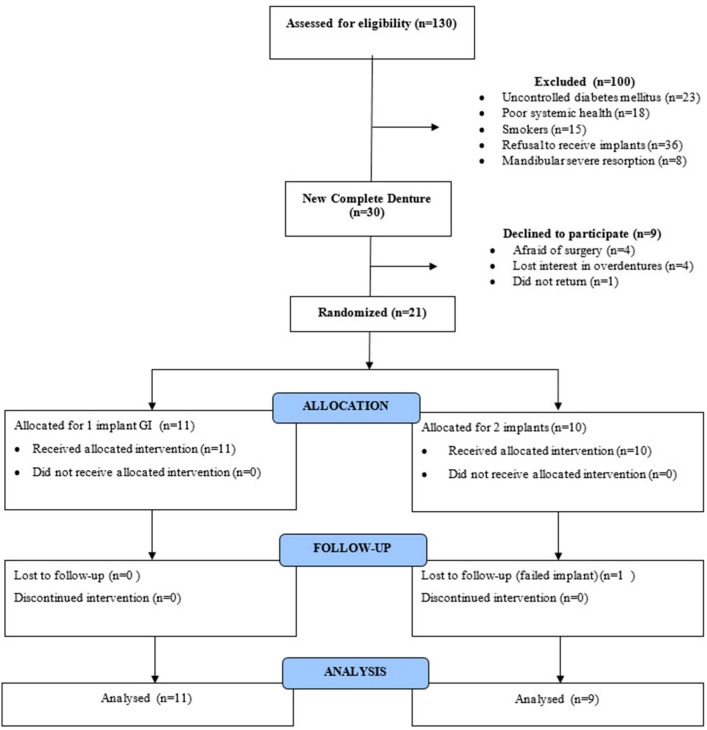
Flowchart of participants

One hundred and thirty individuals were evaluated (82 women and 48 men; mean age: 65±10.2 years). After clinical and radiographic examinations, 100 individuals did not meet the inclusion criteria ( Figure 1 ). Then, 30 individuals were invited to participate in this clinical trial, and each signed an informed consent form prior to enrollment.

### Study interventions and randomization

Maxillary and mandibular CDs were made for 30 individuals under the supervision of two previously trained researchers according to concepts of bilateral balanced occlusion and to the conventional technique previously described ^18^ ^,^ ^19^ . After insertion of the new CDs, the patients were given 30 days before the start of the experimental procedures; this was necessary for functional adaptation and adjustment of the prosthesis ^18^ ^,^ ^19^ .

After the adaptation period, nine individuals withdrew the consent to participate ( Figure 1 ). A baseline assessment was performed on the remaining participants. It included an evaluation of patient satisfaction and masticatory performance based on their new conventional CDs. Later, the twenty-one participants were randomly distributed to receive one (GI, n = 11) or two (GII, n = 10) implants in the mandibular arch. The randomization into different groups was determined using computer-generated numbers (BioEstat - Universidade Federal do Pará; Belém, Pará, Brazil), which were the responsibility of a single researcher. Each participant initially received a number and later those numbers were drawn randomly for each of the groups evaluated. On the day of the surgery, the surgeon was informed of the number of implants to be received by each participant.

**Figure 2:**
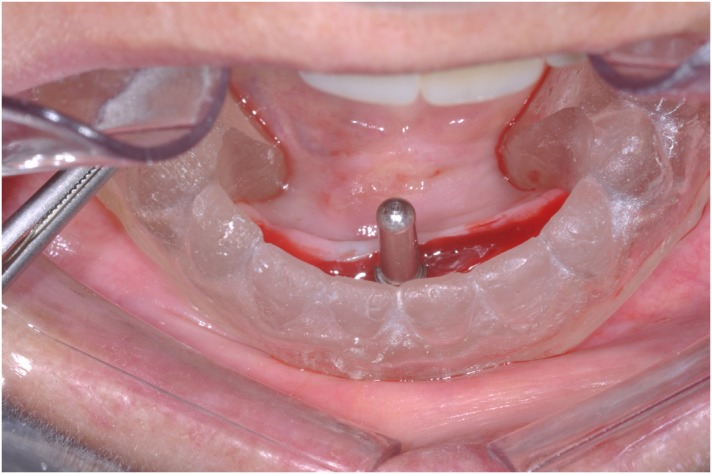
Implant positioner and surgical guide (GI)

**Figure 3:**
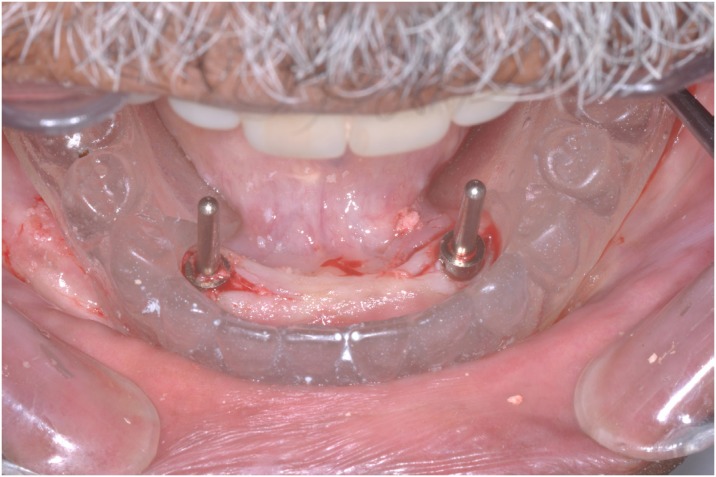
Implant positioner and surgical guide (GII)

Patients’ mandibular CDs were duplicated in cold curing clear acrylic resin material to produce a surgical guide. Surgical procedures included a conventional loading protocol, as described by Cordioli, et al. ^10^ (1997). Single implants (Conexão Sistema de Prótese Ltda; Arujá, São Paulo, Brazil), 3.75 mm diameter and 11.5 mm length, were inserted into the mandibular midline in participants assigned to GI, while two implants were inserted bilaterally into the canine areas in participants assigned to GII ( Figures 2 and 3 ). Surgical procedures were performed by a surgeon, specialist in implantology. After suturing, denture bases were relieved in the implant area and relined with a soft acrylic temporary relining material (Dentusoft - Densell; Buenos Aires, Buenos Aires, Argentina). Postoperative care included oral analgesics and antibiotics and a daily mouth rinse with 0.12% chlorhexidine.

**Figure 4:**
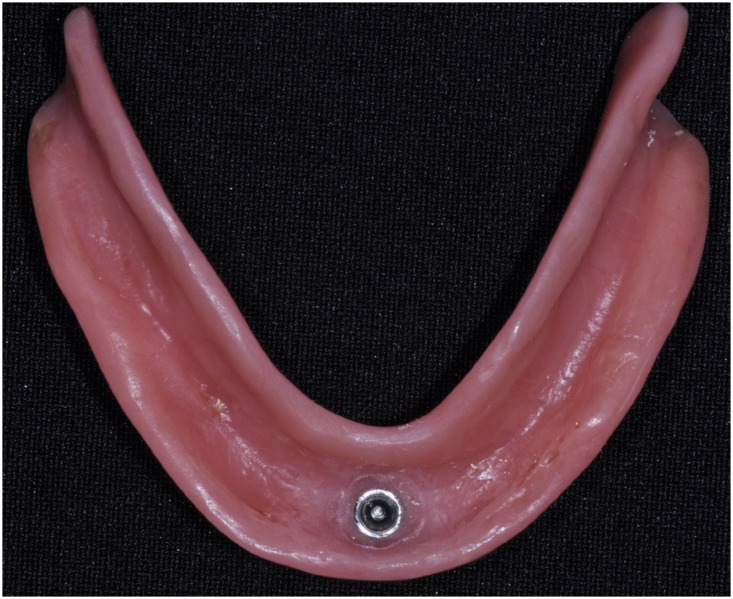
Mandibular overdenture supported by one implant

**Figure 5:**
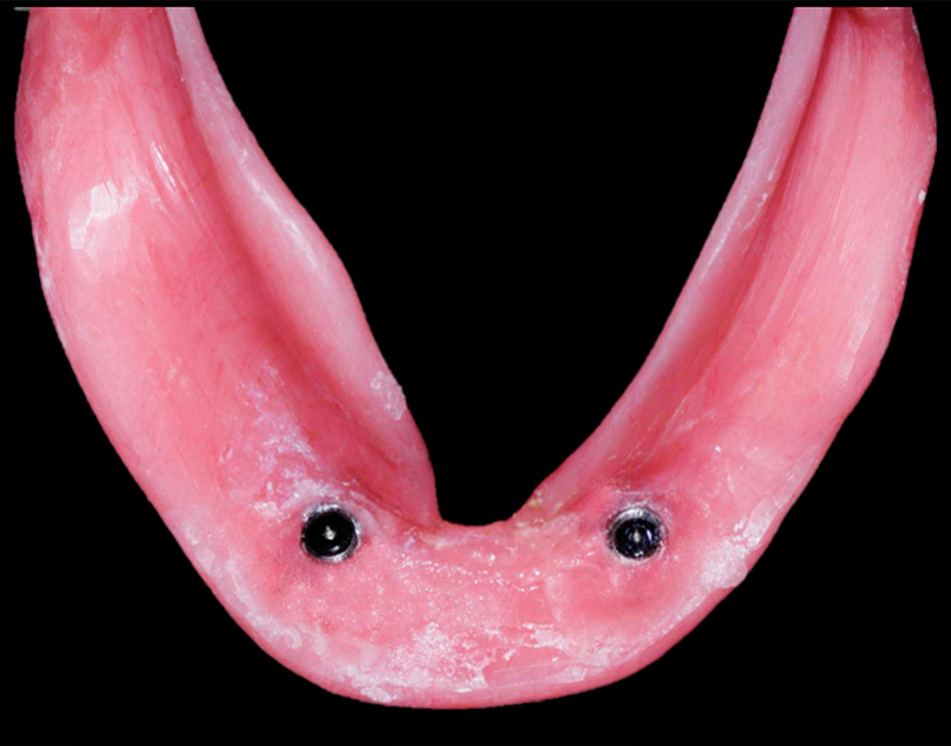
Mandibular overdenture supported by two implants

The second surgical stage was carried out four months after implant placement. Healing abutments were inserted into the implants, and denture bases were relieved and relined. After one to two weeks, o-ring abutments (Conexão Sistema de Prótese Ltda; Arujá, São Paulo, Brazil) were used as overdenture retainers and CDs were relieved and adjusted to engage the matrix attachments. Matrices were polymerized into the denture base with a cold curing acrylic resin (Jet - Clássico; São Paulo, São Paulo, Brazil) ^8^ ^,^ ^10^ ^-^ ^12^ . During polymerization of the resin, the patient was asked to keep his/her denture in centric occlusion using moderate pressure, so that the denture base was in intimate contact with the supporting tissues ^11^ ( Figures 4 and 5 ). Prosthodontic procedures were performed by graduate students under supervision of two professors. Recall appointments were scheduled for 1 week after the procedure, and patients were then referred for follow-up evaluations 3, 6, and 12 months after the delivery of the MOD. Satisfaction with their dentures (maxillary CD and MOD) and masticatory performance were assessed in each follow-up visit by a different examiner.

### Study outcomes

**Figure 6:**
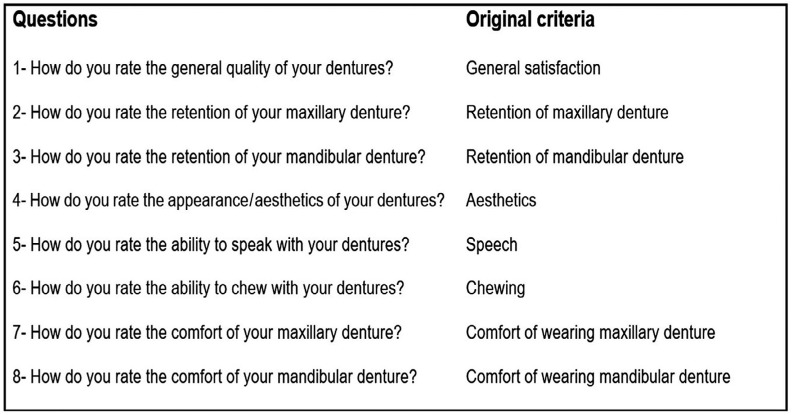
Questions of the satisfaction questionnaire

Satisfaction with dentures was assessed by a questionnaire ( Figure 6 ) used in previous trials ^19^ ^,^ ^20^ . The questionnaire considered eight items, as follows: comfort in wearing mandibular denture; comfort in wearing maxillary denture; retention of maxillary denture; retention of mandibular denture; aesthetics; speech; chewing; and general satisfaction. Possible answers for each question and respective scores were (A) unsatisfactory (“0”), (B) regular (“1”), or (C) good (“2”). For each evaluation, the sum of scores could range from 0 to 16. The questionnaire was applied by another researcher who had been blinded to the previous procedures involved in this trial.

Masticatory performance was assessed by one researcher also blinded to the previous procedures using almonds as natural test food ^18^ ^,^ ^21^ . Participants were invited to chew five almonds deliberately through 20 chewing strokes ^18^ ^,^ ^21^ . A single calibrated operator counted the cycles and collected the comminuted particles in a container.

Each participant received 50 ml of water to rinse the mouth and remove remaining particles, which were put away in a container. Contents were then poured into a sieve (sieve 1-7 cm, dim. 175×78×40 mm) adapted to a filter paper to separate liquid and chewed material. Five-hundred ml of water were dumped on the sieve to eliminate saliva present in almonds and reduce particle clumping.

The crushed almonds were dehydrated in an electric oven (Fanem; Guarulhos, São Paulo, Brazil) at a controlled temperature of 130°C for 40 minutes. The content was subjected to a 4-sieve series in a gypsum vibrator, under constant vibration for 60 seconds. The sieves used (Granutest-Telastem; Bom Retiro, São Paulo, Brazil) were approved by the ABNT - Brazilian Association of Technical Standards, and had different hole sizes: 4.0 mm (ABNT 5), 2.8 mm (ABNT 7), 2.0 mm (ABNT 10) and 1.0 mm (ABNT 18).

After dehydration, the crushed almonds were weighed on a precision balance (Ind. e Com. Eletrónica Gehaka Ltda.; São Paulo, São Paulo, Brazil) and the values were recorded. The masticatory performance was calculated as the weight of comminuted material passed through the 2.8 mm sieve. From these values, the masticatory performance was calculated based on the index proposed by Kapur and Soman ^21^ (2004):

MP=P1×100÷Pt

where:

MP means masticatory performance (in percentage);

P1 represents the material weight sum in sieves 3 and 4 and background collector;

Pt is the value corresponding to the total material weight subjected to sieving.

### Sample size and statistical analysis

The sample size was calculated considering a previous pilot study, in which a convenience sample of 7 patients underwent the proposed protocol. This study considered the variable “masticatory efficiency” as the primary factor. The minimum significant difference considered for this variable was 17 and a standard deviation of 15.2 was obtained. Considering a power analysis of 80% and α=0.05, a total of 14 individuals was determined for each group.

Data from the satisfaction questionnaire were analyzed using the Friedman test to compare different periods (baseline, 3, 6, and 12 months) within groups. The Mann-Whitney U test was used to make comparisons between the groups. Data on masticatory performance were assessed using the oneway repeated measures analysis of variance (ANOVA). The Bonferroni correction for multiple comparisons of means was used to identify when the masticatory performance varied significantly (at baseline, 3, 6, or 12 months). The independent samples t-test was used to make comparisons between two groups. All comparisons were performed with a significance level of 5%. Statistical tests were conducted using the PASW Statistics software version 19 (SPSS Inc.; Chicago, Illinois, USA).

## Results

Table 1 shows baseline demographic and clinical characteristics for each group and Figure 1 shows a flowchart of participants. It was observed that after the conventional CDs were delivered, nine individuals withdrew from the research. However, the withdrawals happened before baseline evaluation and randomization. During the follow-up, only one participant in GII declined to participate.

**Table 1:** Baseline characteristics of patients in both groups

Characteristic	Group I	Group II
	Mean (±SD * )	Mean (±SD * )
Age (years)	64.4 (±8.3)	64 (±6.4)
Gender (male/female)	05/06	05/05
Mandibular bone height in the symphyseal area (mm)	16.6 (1.5)	16.7(1.3)

* SD: standard deviation

Two implants were lost during this trial. The first failure occurred in a male patient from GI (1/11) during the insertion of healing abutments (four months after the surgery). In this case, a new implant was installed after the patient healed, and he remained in the trial. The other failure occurred in a female patient from GII (1/20) three months after the surgery. The patient refused to receive a new implant and declined to continue with the trial. Hence, a total average for an implant survival rate of 93.5% was obtained (90.9% for GI and 95% for GII). Two midline fractures in denture (1 for GI and in 1 for GII) were found during the follow-up.

**Figure 7:**
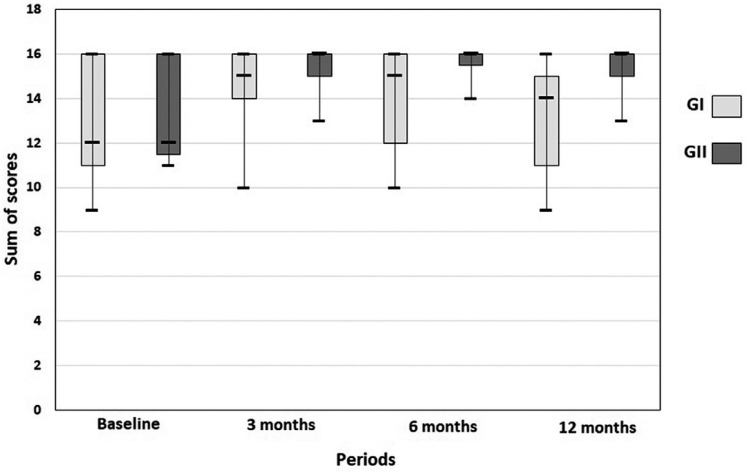
Overall patient satisfaction for GI and GII

Figure 7 shows a significant improvement in the overall patient satisfaction for GI in periods of 3 (p=0.02) and 6 months (p = 0.04) relative to baseline (conventional CDs). At 12 months, the patient satisfaction became similar to baseline (p=0.74). Satisfaction levels were similar between 3 and 6 months (p=0.74). GII also had a significant improvement (p<0.05) in overall patient satisfaction for all periods compared to baseline. There was no difference (p>0.05) between all periods evaluated after implant placement. Finally, when groups were compared to each other ( Table 2 ), GI had similar levels of satisfaction to those of GII at 3 and 6 months. The only difference was observed at 12 months, when satisfaction was higher among GII participants (p=0.01).

**Table 2:** Overall patient satisfaction regarding mandibular overdentures supported by one (GI) or two (GII) implants. Comparison between groups (mean±standard deviation)

Group	Baseline	3 months	6 months	12 months
GI	13±2.36	14.36±2.11	14.18±2.13	13.36±2.2
GII	13.33±2.17	15.44±1.13	15.67±0.7	15.44±1.01
	p=0.784	p=0.18	p=0.08	p=0.01 *

* significant difference between groups (p<0.05) Minimum possible value: 0; maximum possible value: 16

Table 3 represents the comparison between groups considering each question of the questionnaire. The satisfaction with the retention of mandibular denture was lower (p=0.006) in GI in comparison to GII at 12 months. The other questions demonstrated similar results between both groups.

**Table 3:** Values for questions of the satisfaction questionnaire. Comparison between groups (mean±standard deviation)

Period	Baseline			3 Months			6 Months			12 months		
Group	GI	GII	p-value	GI	GII	p-value	GI	GII	p-value	GI	GII	p-value
Q1	1.9±0.3	1.77±0.44	0.62	2±0	2±0	1	1.63±0.5	2±0	0.17	1.63±0.5	2±0	0.17
Q2	2±0	2±0	1	1.72±0.46	1.89±0.33	0.54	1.81±0.4	1.88±0.33	0.79	1.63±0.5	1.88±0.33	1.34
Q3	0.9±0.83	1.33±0.5	0.25	1.72±0.46	2±0	0.3	1.63±0.5	2±0	0.17	1.27±0.46	2±0	0.006 *
Q4	2±0	1.77±0.44	0.4	1.81±0.4	1.89± 0.33	0.79	1.81±0.4	2±0	0.49	1.9±0.3	2±0	0.73
Q5	1.81±0.4	1.66±0.5	0.56	1.9±0.3	1.89±0.33	0.93	2±0	2±0	1	1.72±0.46	2±0	0.3
Q6	1.36±0.8	1.33±0.5	0.73	1.63±0.67	1.89±0.33	0.51	1.63±0.5	1.77±0.44	0.59	1.72±0.46	1.88±0.33	0.54
Q7	1.81±0.4	2±0	0.49	1.81±0.4	2±0	0.49	1.9±0.3	2±0	0.73	1.9±0.3	1.88±0.33	0.93
Q8	1.18±0.75	1.44±0.52	0.49	1.72±0.64	1.88±0.33	0.76	1.72±0.46	2±0	0.3	1.54±0.52	1.77±0.44	0.38

* significant difference between groups (p<0.05) Minimum possible value: 0; maximum possible value: 2

**Figure 8:**
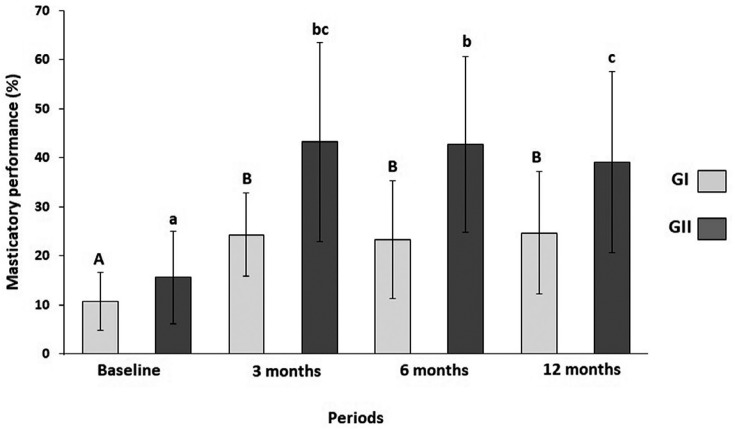
Masticatory performance among GI and GII participants (%)

Figure 8 shows a significant increase (p<0.05) in the masticatory performance among GI participants in all periods related to baseline. There was no difference (p>0.05) among periods evaluated after implant placement. A significant increase in the masticatory performance was also observed for GII in all periods related to baseline ( Figure 8 ). At 12 months, patients’ reports showed a lower masticatory performance compared to 6 months (p=0.46) and results were similar to those from 3 months (p=0.511). When groups were compared to each other ( Table 4 ), GI participants’ reports showed a lower masticatory performance (p<0.05) compared to GII participants in all periods evaluated.

**Table 4:** Masticatory performance (%) among patients with mandibular overdentures supported by one (GI) or two (GII) implants. Comparison between the groups (mean±standard deviation)

Group	Baseline	3 months	6 months	12 months
GI	10.7±6.1	24.3±8.5	23.3±12	24.7±12.5
GII	15.6±9.4	43.2±20.2	42.6±17.9	39.14±18.4
	p=0.20	p=0.02 *	p=0.01 *	p=0.04 *

* significant difference between groups (p<0.05)

## Discussion

Considering the findings of this study, the null hypothesis was rejected because there were significant differences after one year between and within both groups in most parameters evaluated.

Based on results obtained from GI at 3 and 6 months, the overall satisfaction with the dentures for this group was expected to remain high for an entire year. However, at 12 months, participants in this group experienced a decrease in satisfaction compared to prior periods and to GII. These findings contrast with other prospective studies ^5^ ^,^ ^9^ ^,^ ^14^ . Tavakolizadeh, et al. ^14^ (2015) observed an improvement in satisfaction among patients with MOD supported by one or two implants after six and twelve months when these patients were compared to others with conventional CDs. The authors also reported that both groups had similar results in the periods evaluated. In a five-year prospective study, Bryant, Walton and MacEntee ^9^ (2015) also found similar satisfaction levels among subjects treated with MOD supported by one or two implants, and the results of these two groups were always higher than baseline levels among participants with conventional CDs. Kronstrom, et al. ^5^ (2017), in a prospective study of five years, stated that patient satisfaction scores in MOD supported by one or two implants increased significantly when compared to baseline values and remained high for both groups, with no significant differences.

In our study, the lower overall satisfaction levels of GI participants at 12 months may be associated to a perception of a lower retention (p=0.006) of their MOD in comparison to the GII, as shown in Table 3 . This difference was not observed in other evaluated periods.

The satisfaction of patients with their dentures may be also influenced by the attachment system for implant-retained overdentures, since patients have a strong preference for more retentive attachments ^22^ . In this study, the attachment system used had a metal abutment and a metallic matrix with a retentive ring made of rubber nitryl (Conexão Sistema de Prótese Ltda; Arujá, São Paulo, Brazil). Botega, et al. ^23^ (2004) evaluated the o-ring system and found a retention force ranging from 12.7 N to 14.4 N. Similarly, da Fontoura Frasca, et al. ^24^ (2014) reported that values for retention force ranged from 11.75 N to 13.7 for the o-ring system, while the ball attachment had higher values, which ranged from 42.7 to 44.7 N. Most studies that observed continuity of high levels of patient satisfaction used a ball attachment for implant-retained overdentures ^5^ ^,^ ^9^ ^,^ ^14^ . Although the retention force of o-ring system was not measured in this study, it may be suggested, based on literature data ^23^ ^,^ ^24^ , that the other previous studies ^5^ ^,^ ^9^ ^,^ ^14^ used retention systems with higher retention force than that one used in this study and, consequently, showed better results for subjects treated with MOD supported by one implant.

It is also important to mention that new maxillary and mandibular CDs were produced for all participants to normalize the aesthetic and functional parameters of their dentures. The production of new CDs is a step that has also been used in other studies ^1^ ^,^ ^5^ ^,^ ^15^ . Probably, this fact was responsible for high levels of satisfaction in the baseline. It is possible to assume that if patients were wearing their old CDs instead of the new ones, the baseline would be lower and the improvement in the satisfaction levels would be more evident.

In this study, the results showed that treatment with MOD supported by one implant improved patients’ masticatory performance (p<0.05) when results were compared to treatment with CDs, and this improvement remained constant for one year. These results are in accordance with those obtained by Grover, Vaidyanathan and Veeravalli ^1^ (2014), who observed improvement in the masticatory performance in the first six months among patients treated with MOD supported by one implant. Harder, et al. ^11^ (2011) and Cheng, et al. ^15^ (2012) also reported similar results after four weeks and immediately after attachment placement in patients with MOD supported by one implant.

GII participants exhibited average masticatory performance scores that were better than those exhibited by GI participants (mean values of 41.6%±18.2 and 24.1%±10.8, respectively). This difference (p<0.05) may be explained by the higher retention of the mandibular overdenture perceived by wearers of MOD supported by two implants. The second implant used for MOD retention promoted a greater retention of the overdenture and improved the patient's self-confidence when chewing. Nevertheless, the lower retention of MOD supported by one implant and the lower resistance against horizontal movements may lead to less denture stability during chewing and to a reduced masticatory performance.

Another hypothesis that may have also contributed to the significant differences in masticatory performance between the two groups is the potential rotational motion of overdentures supported by a single implant during mastication. One of the main concerns regarding overdentures supported by one implant refers to the movement of the prosthesis around the central axis. Krennmair, et al. ^25^ (2001), for example, affirmed that one disadvantage of overdentures supported by a single implant is the development of sagittal, transverse, and vertical rotational axes and implant position. However, this problem can be minimized during prosthetic treatment by increasing lateral (sublingual) extensions in the prosthesis, when possible.

Alqutaibi, et al. ^8^ (2017) observed that MOD supported by two implants may promote a greater improvement in the muscle activity of completely edentulous patients if compared to the single implant. Consequently, muscle activity was more devoted to and directed towards masticatory function and less effort was required to stabilize or retain the prosthesis ^8^ . This could also be a possible explanation for the best results found in GII for masticatory performance.

A total average for an implant survival rate of 93.5% was obtained (90.9% for GI and 95% for GII). These results are consistent with those reported by Alsabeeha, et al. ^26^ (2011), who found a survival rate of 91.7% twelve months after installation of 36 single implants in mandibles. Our failure rates were higher than those reported by Harder, et al. ^11^ (2011), Liddelow and Henry ^12^ (2010), and Passia, Wolfart and Kern ^27^ (2015), all of whom obtained an implant survival rate of 100% after different follow-up periods.

The needs for prosthetic maintenance were higher if compared to other studies ^9^ ^,^ ^10^ ^,^ ^12^ . Some unscheduled appointments were needed and associated to the repair of fractured overdentures, as two midline denture fractures were found for GI and in one for GII. Gonda, et al. ^28^ (2010) also observed 21.4% and 9.3% of fractures of denture base in overdentures retained by one or two implants, respectively. Osman, et al. ^29^ (2014) suggested that a higher rate of fractures of overdenture must be expected if the denture base is not reinforced, particularly if the size of the matrix attachment is relatively large. In a systematic review, Assaf, et al. ^30^ (2017) evaluated the types of maintenance and complications in MOD with a variable number of implants and found evidence that a mean complication rate cannot be determined due to the multiplicity of contributing factors.

The main limitation of this study is the small number of participants. Considering the strict inclusion and exclusion criteria, most patients evaluated were not included in this research. Consequently, this sample size limits the generalizability of this study. The low number of participants can influence the power analysis of this research, estimated at 80%, which could have been higher if there were more participants.

Further studies are needed to clinically evaluate other aspects involved in the treatment of edentulous patients with MOD supported by one implant, and should include an assessment of the effectiveness and durability of different attachment systems. Finally, more long-term follow-up studies on this subject with a larger number of participants are also recommended.

## Conclusions

Within the limitations of this one-year prospective study, it is possible to conclude that:

The replacement of conventional mandibular CDs with MOD improves the masticatory performance, regardless of the number of implants;

MOD supported by two implants had better masticatory performance than MOD supported by one implant;

MOD supported by one implant resulted in similar levels of patient satisfaction at 3 and 6 months, but lower patient satisfaction at 12 months in comparison to MOD supported by two implants.

## References

[1] Grover M, Vaidyanathan AK, Veeravalli PT (2014). OHRQoL, masticatory performance and crestal bone loss with single-implant, magnet-retained mandibular overdentures with conventional and shortened dental arch. Clin Oral Implants Res..

[2] MacEntee MI, Feine JS, Carlsson GE (2003). The impact of edentulism on function and quality of life. Implant overdentures: the standard of care for edentulous patients.

[3] Feine JS, Carlsson GE, Awad MA, Chehade A, Duncan WJ, Gizani S (2002). The McGill consensus statement on overdentures. Mandibular two-implant overdentures as first choice standard of care for edentulous patients.

[4] Fitzpatrick B (2006). Standard of care for the edentulous mandible: a systematic review. J Prosthet Dent.

[5] Kronstrom M, Davis B, Loney R, Gerrow J, Hollender L (2017). Satisfaction and clinical outcomes among patients with immediately loaded mandibular overdentures supported by one or two dental implants: results of a 5-year prospective randomized clinical trial. Int J Oral Maxillofac Implants.

[6] Srinivasan M, Makarov NA, Herrmann FR, Müller F (2016). Implant survival in 1- versus 2-implant mandibular overdentures: a systematic review and meta-analysis. Clin Oral Implants Res.

[7] Elsyad MA (2016). Patient satisfaction and prosthetic aspects with miniimplants retained mandibular overdentures. A 5-year prospective study. Clin Oral Implants Res.

[8] Alqutaibi AY, Kaddah AF, Farouk M (2017). Randomized study on the effect of single-implant versus two-implant retained overdentures on implant loss and muscle activity: a 12-month follow-up report. Int J Oral Maxillofac Surg.

[9] Bryant SR, Walton JN, MacEntee MI (2015). A 5-year randomized trial to compare 1 or 2 implants for implant overdentures. J Dent Res.

[10] Cordioli G, Majzoub Z, Castagna S (1997). Mandibular overdentures anchored to single implants: a five-year prospective study. J Prosthet Dent.

[11] Harder S, Wolfart S, Egert C, Kern M (2011). Three-year clinical outcome of single implant-retained mandibular overdentures - results of preliminary prospective study. J Dent.

[12] Liddelow G, Henry P (2010). The immediately loaded single implant-retained mandibular overdenture: a 36-month prospective study. Int J Prosthodont.

[13] Nogueira TE, Esfandiari S, Leles CR (2016). Cost-effectiveness analysis of the single-implant mandibular overdenture versus conventional complete denture: study protocol for a randomized controlled trial. Trials.

[14] Tavakolizadeh S, Vafaee F, Khoshhal M, Ebrahimzadeh Z (2015). Comparison of marginal bone loss and patient satisfaction in single and double-implant assisted mandibular overdenture by immediate loading. J Adv Prosthodont.

[15] Cheng T, Sun G, Huo J, He X, Wang Y, Ren YF (2012). Patient satisfaction and masticatory efficiency of single implant-retained mandibular overdentures using the stud and magnetic attachments. J Dent.

[16] Bhat S, Chowdhary R, Mahoorkar S (2016). Comparison of masticatory efficiency, patient satisfaction for single, two, and three implants supported overdenture in the same patient: a pilot study. J Indian Prosthodont Soc.

[17] Boven GC, Raghoebar GM, Vissink A, Meijer HJ (2015). Improving masticatory performance, bite force, nutritional state and patient's satisfaction with implant overdentures: a systematic review of the literature. J Oral Rehabil.

[18] Oliveira NM, Rodriguez LS, Mendoza Marin DO, Paleari AG, Pero AC, Compagnoni MA (2014). Masticatory performance of complete denture wearers after using two adhesives: a crossover randomized clinical trial. J Prosthet Dent.

[19] Paleari AG, Marra J, Rodriguez LS, De Souza RF, Pero AC, Mollo FA (2012). A cross-over randomised clinical trial of eccentric occlusion in complete dentures. J Oral Rehabil.

[20] Celebić A, Knezović-Zlatarić D (2003). A comparison of patient's satisfaction between complete and partial removable denture wearers. J Dent.

[21] Kapur KK, Soman SD (2004). Masticatory performance and efficiency in denture wearers. 1964. J Prosthet Dent.

[22] Trakas T, Michalakis K, Kang K, Hirayama H (2006). Attachment systems for implant retained overdentures: a literature review. Implant Dent.

[23] Botega DM, Mesquita MF, Henriques GE, Vaz LG (2004). Retention force and fatigue strength of overdenture attachment systems. J Oral Rehabil.

[24] Fontoura Frasca LC, Castro Mattia PR, Botega DM, Rivaldo EG (2014). Evaluation of retention forces and resistance to fatigue of attachment systems for overdentures: plastic and metal components. Implant Dent.

[25] Krennmair G, Ulm C (2001). The symphyseal single-tooth implant for anchorage of a mandibular complete denture in geriatric patients: a clinical report. Int J Oral Maxillofac Implants.

[26] Alsabeeha NH, Payne AG, De Silva RK, Thomson WM (2011). Mandibular single-implant overdentures: preliminary results of a randomised-control trial on early loading with different implant diameters and attachment systems. Clin Oral Implants Res.

[27] Passia N, Wolfart S, Kern M (2015). Six-year clinical outcome of single implant-retained mandibular overdentures - a pilot study. Clin Oral Implants Res.

[28] Gonda T, Maeda Y, Walton JN, MacEntee MI (2010). Fracture incidence in mandibular overdentures retained by one or two implants. J Prosthet Dent.

[29] Osman RB, Ma S (2014). Prosthodontic maintenance of overdentures on zirconia implants: 1-year results of a randomized controlled trial. Int J Prosthodont.

[30] Assaf A, Daas M, Boittin A, Eid N, Postaire M (2017). Prosthetic maintenance of different mandibular implant overdentures: a systematic review. J Prosthet Dent.

